# Dissecting the association between gut microbiota and hypertrophic scarring: a bidirectional Mendelian randomization study

**DOI:** 10.3389/fmicb.2024.1345717

**Published:** 2024-03-21

**Authors:** Kaikai Xue, Guojian Zhang, Zihao Li, Xiangtao Zeng, Zi Li, Fulin Wang, Xingxing Zhang, Cai Lin, Cong Mao

**Affiliations:** ^1^Key Laboratory of Orthopedics of Zhejiang Province, Department of Orthopedics, The Second Affiliated Hospital and Yuying Children’s Hospital of Wenzhou Medical University, Wenzhou, China; ^2^Department of Burn, The First Affiliated Hospital of Wenzhou Medical University, Wenzhou, China; ^3^Department of Endocrinology and Metabolism, The First Affiliated Hospital of Wenzhou Medical University, Wenzhou, China

**Keywords:** gut microbiota, hypertrophic scar, *Firmicutes*, Mendelian randomization, GWAS

## Abstract

Hypertrophic scars affect a significant number of individuals annually, giving rise to both cosmetic concerns and functional impairments. Prior research has established that an imbalance in the composition of gut microbes, termed microbial dysbiosis, can initiate the progression of various diseases through the intricate interplay between gut microbiota and the host. However, the precise nature of the causal link between gut microbiota and hypertrophic scarring remains uncertain. In this study, after compiling summary data from genome-wide association studies (GWAS) involving 418 instances of gut microbiota and hypertrophic scarring, we conducted a bidirectional Mendelian randomization (MR) to investigate the potential existence of a causal relationship between gut microbiota and the development of hypertrophic scar and to discern the directionality of causation. By utilizing MR analysis, we identified seven causal associations between gut microbiome and hypertrophic scarring, involving one positive and six negative causal directions. Among them, *Intestinimonas*, *Ruminococcus2*, *Barnesiella*, *Dorea*, *Desulfovibrio piger*, and *Ruminococcus torques* act as protective factors against hypertrophic scarring, while *Eubacterium rectale* suggests a potential role as a risk factor for hypertrophic scars. Additionally, sensitivity analyses of these results revealed no indications of heterogeneity or pleiotropy. The findings of our MR study suggest a potential causative link between gut microbiota and hypertrophic scarring, opening up new ways for future mechanistic research and the exploration of nanobiotechnology therapies for skin disorders.

## 1 Introduction

With an estimated incidence reaching up to 70%, hypertrophic scar stands as one of the prevalent complications in burn patients, characterized by abnormal, elevated, and thickened tissue growth at the site of healed skin lesions (Bombaro et al., 2003). Various factors contribute to the development and progression of hypertrophic scar, with local risk variables such as wound or scar stress, systemic factors like hypertension, genetic factors including single-nucleotide polymorphisms, and lifestyle choices all playing a role. To address hypertrophic scar volume and alleviate discomfort and itch of patients, a suitable approach is crucial. Clinical available interventions include surgery, radiotherapy, and conservative treatments such as gel sheets, tape fixation, and the application of topical or injectable external medications, should be utilized on an individual basis (Ogawa, 2022), as their improper use can potentially lead to adverse effects such as skin shrinkage, telangiectasia, pigmentation issues, skin ulcers, and other imperfections (Lee and Jang, 2018). The impact of hypertrophic scars places a considerable psychological and financial burden on affected patients. Consequently, it is imperative to delve into a comprehensive understanding of the modifiable risk factors and the potential ramifications associated with hypertrophic scarring.

The human gut microbiota is characterized by a rich diversity of bacteria, comprising up to 1,000 different microbial species (Li et al., 2022). This intricate microbial community plays a pivotal role in a myriad of physiological functions, exerting a profound influence on human health. Dysbiosis of the gut microbiota, including both compositional and functional imbalances, is associated with a spectrum of diseases ranging from local gastrointestinal conditions to neurological, metabolic, hepatic, and cardiovascular diseases (Lynch and Pedersen, 2016). Actually, due to the heterogeneity in experimental procedures and study designs, it is currently challenging to identify distinct microbiome signatures for most human diseases, including some intestinal diseases such as pediatric celiac disease (where environmental factors during childhood may not be as significant as in adulthood; Abdukhakimova et al., 2021). However, certain specific bacteria within the microbiota may warrant further investigation to understand their potential applications as probiotic therapies, diagnostic tools, or prognostic biomarkers. The emerging concept of the gut–skin axis outlines the interaction between gut microbiota and skin (Saarialho-Kere, 2004; Salem et al., 2018; Mahmud et al., 2022). More specifically, the health and balance of gut microbiota can influence the condition and function of skin, and vice versa. The effects induced by microbiota on specific inflammatory skin disorders are thought to originate from factors such as a compromised intestinal barrier, elevated levels of inflammatory mediators, and the release of metabolites by microbes (Mahmud et al., 2022). Nevertheless, the presence of a causal relationship between gut microbiota and hypertrophic scarring remains uncertain. Therefore, it is crucial to thoroughly explore this potential causal link.

Mendelian randomization (MR) is a statistical technique employed to assess causal relationships between a risk factor or biomarker (exposure) and a disease (outcome) by leveraging genetic variations as instrumental variables. The fundamental principle underlying MR is to utilize the random assortment of genetic material during meiosis, the process of gamete formation, to simulate a randomized controlled trial and draw inferences regarding causation (Burgess et al., 2015). MR presents distinct advantages over conventional observational research by providing more robust evidence for causal relationships. This strength arises from the fact that genetic variations are established at conception, thereby minimizing susceptibility to confounding or reverse causation (Abdellaoui et al., 2023).

In this work, we aim to explore the causal connections between gut microbiota and hypertrophic scarring and identify specific gut microbiota through a bidirectional MR analysis. The findings of this study may provide valuable insights for future research on the genetic underpinnings and biological therapies related to the intricate features associated with gut microbiota and hypertrophic scar.

## 2 Materials and methods

### 2.1 Study design and ethics statement

**Figure 1:**
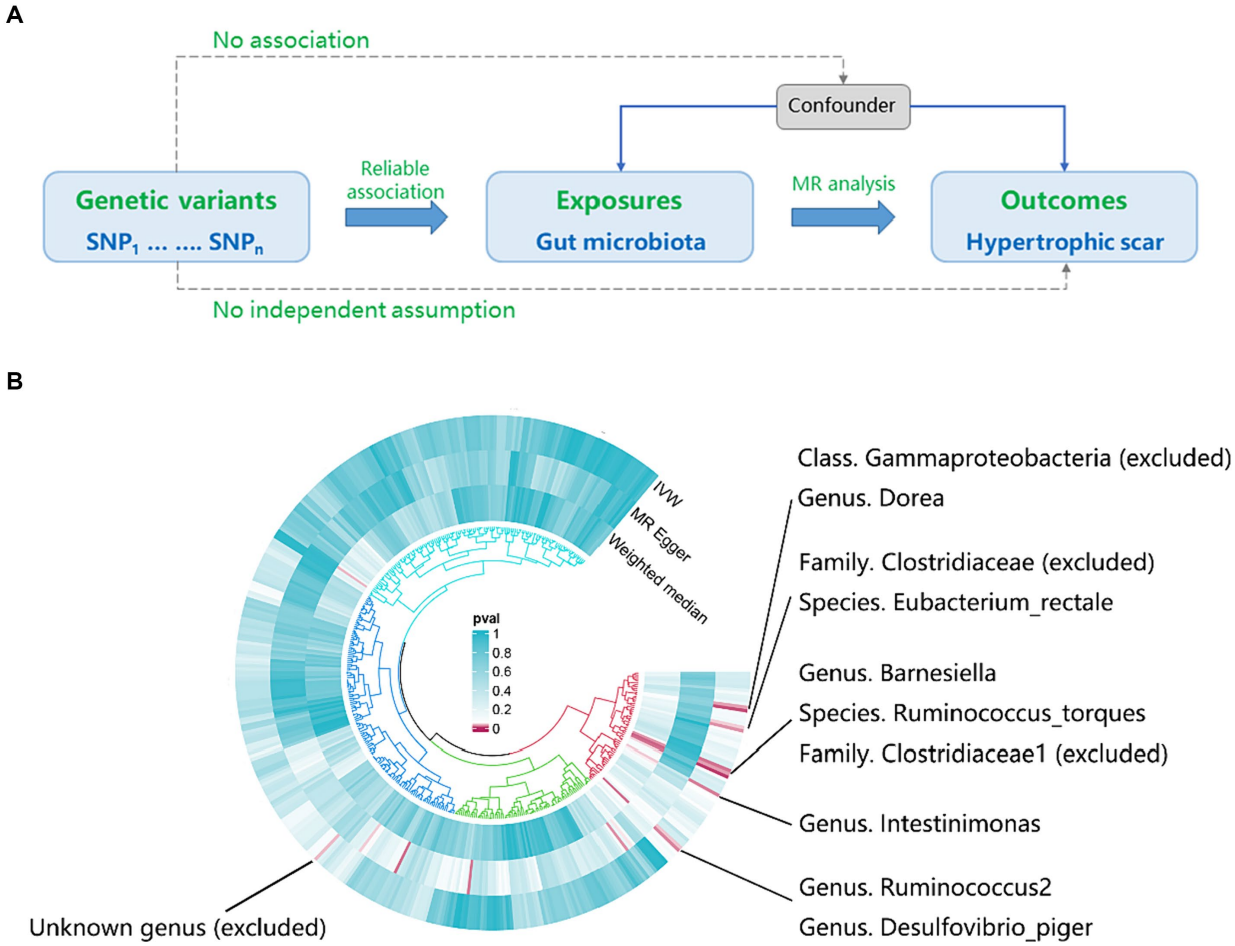
The workflow and initial results of a two-sample Mendelian Randomization (MR). **(A)** Fundamental analysis flow for MR; **(B)** Identified gut microbiota associated with hypertrophic scarring through initial MR analysis (*p*_IVW_ < 0.05), and the exclusions of unknown genera and gut microbiota that did not meet the specified criteria of instrumental variable selection.

Figure 1A provides a concise overview of the fundamental analysis flow. Employing genome-wide association study (GWAS) summary statistics, we performed a bidirectional two-sample MR to investigate the causal link between gut microbiota (exposure) and hypertrophic scarring (outcome). The MR design hinges on three critical assumptions. Firstly, the genetic variants should demonstrate a reliable association with the exposure. Secondly, the genetic variants should not be correlated with any confounders affecting the relationship between the exposure and the outcome. Lastly, these genetic variants must link to the outcome exclusively through the exposure.

Our analyses utilized publicly available GWAS data, eliminating the need for ethics committee approval.

### 2.2 Data sources

The GWAS summary data for 418 gut microbiotas were obtained from the MiBioGen consortium (Kurilshikov et al., 2021) and the Dutch Microbiome Project (Lopera-Maya et al., 2022). The MiBioGen consortium meticulously curated and examined genome-wide genotypes in conjunction with 16S fecal microbiome data sourced from a vast pool of 18,340 European populations. Additionally, the Dutch Microbiome Project undertook a comprehensive genome-wide association study encompassing 207 taxa and 205 pathways, depicting the intricate landscape of microbial composition and function within a substantial cohort comprising 7,738 participants.

The summary data for the GWAS on hypertrophic scar were sourced from the FinnGen consortium (L12_HYPETROPHICSCAR). This dataset comprised a vast array of information, encompassing 16,380,443 single-nucleotide polymorphisms (SNPs) and a sample size of 208,248 European populations.

All GWAS data used in this study are available in the IEU Open GWAS Project.

### 2.3 Instrumental variable selection

To fortify data robustness and maintain result accuracy, we selected SNPs associated with gut microbiota by using a reasonably comprehensive threshold (*p* < 1 × 10^−5^). This approach aligns with established practices from previous studies, reinforcing the reliability of our candidate SNPs selection (Li Y et al., 2023; Li N et al., 2023; Wang et al., 2023; Zhang Y et al., 2023). Subsequently, we conducted a linkage disequilibrium analysis using PLINK software (v1.9)^1^ to clump SNPs with a linkage disequilibrium distance exceeding 10,000 kb and an r^2^ less than 0.001. PLINK is a powerful and versatile command-line toolset designed for the analysis of genetic data. The CLUMP function is a specific feature within PLINK that is often used for post-GWAS analysis. It is used for clumping together genetic variants based on linkage disequilibrium patterns, helping identify a subset of independent genetic variants that capture the association with the exposure variable. To assess the robustness of our chosen instrumental variables, we employed the F statistic, with a threshold of over 10 being commonly considered indicative of strong instrumental variables, thereby ensuring the reliability of our evaluation (Pierce et al., 2011; Brion et al., 2013). The F-statistic, often used in statistical hypothesis testing, has various applications across different fields. In an MR analysis, it is used to evaluate whether the genetic instrument is strong enough to provide reliable causal inference. A higher F-statistic indicates a stronger instrument. Moreover, our analysis was constrained to results derived from a minimum of three shared SNPs (Li FF et al., 2023).

### 2.4 Statistical analysis

This study primarily employed the inverse-variance weighted (IVW) method to estimate associations between gut microbiota and hypertrophic scarring. The IVW method combines these individual variant-exposure and variant-outcome associations to obtain an overall estimate of the causal effect. It assumes that all genetic variants are valid instruments and that there is no horizontal pleiotropy (i.e., the genetic variants only affect the outcome through their impact on the exposure). While the IVW method is widely used, researchers should also consider sensitivity analyses and other methods, such as weighted median and MR-Egger regression, to assess the robustness of results and detect potential violations of assumptions. Therefore, results from MR were deemed meaningful if the IVW method identified a significant association (*p* < 0.05), and concordantly, two additional methods, MR-Egger regression and weighted median (WM), also indicated effects in the same direction (Li FF et al., 2023).

Sensitivity analyses were evaluated through leave-one-out analysis and the Q-test of both MR Egger and IVW methods (Yang et al., 2023), and directional horizontal pleiotropy was examined using the Egger intercept calculation (Cui and Tian, 2021). Leave-one-out analysis is a technique used to assess the robustness and influence of individual data points in statistical models, systematically removing one genetic instrument at a time and re-evaluating the causal estimates. The Q-test is a statistical test used in MR to assess the presence of heterogeneity among the causal estimates obtained from individual genetic instruments. This test is particularly applied in the IVW and MR Egger methods. Additionally, directional horizontal pleiotropy refers to a situation where genetic variants used as instruments have pleiotropic effects on the outcome, and there is a systematic directional bias. The MR Egger method is designed to address situations where there is directional horizontal pleiotropy by allowing for an intercept in the regression model.

Furthermore, a reverse model was implemented to estimate the effect of hypertrophic scarring on gut microbiota. This analysis aimed to explore the potential existence of a reverse-direction causal relationship, assessing whether the exposure is positioned upstream of the outcome (Li W et al., 2023). Reverse MR is a relatively less common approach compared to standard MR analyses, but it can provide insights into the potential causal relationships between outcomes and exposures, especially in situations where conventional study designs may face challenges.

We performed all statistical analyses using R (version 4.2.2) and TwoSampleMR package (version 0.5.7). The detail analysis process and script have been uploaded to GitHub.^2^

## 3 Results

### 3.1 Causal effects of gut microbiota on hypertrophic scarring

**Figure 2:**
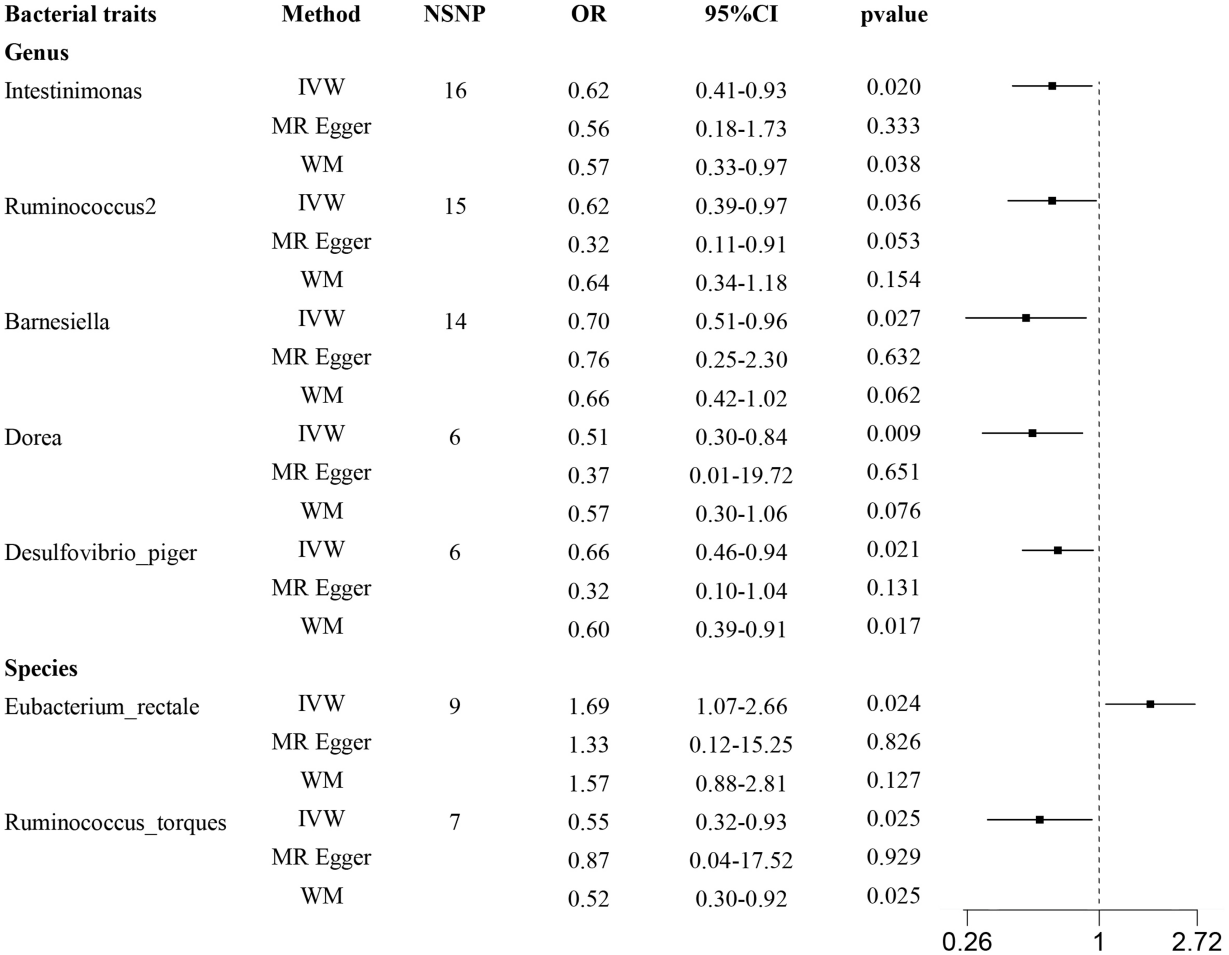
Forest plots show causal-effect estimates of gut microbiota and hypertrophic scarring. IVW, inverse-variance weighted; WM, weighted median; NSNP, the number of SNP; OR, odds ratio; CI, confidence interval.

**Figure 3:**
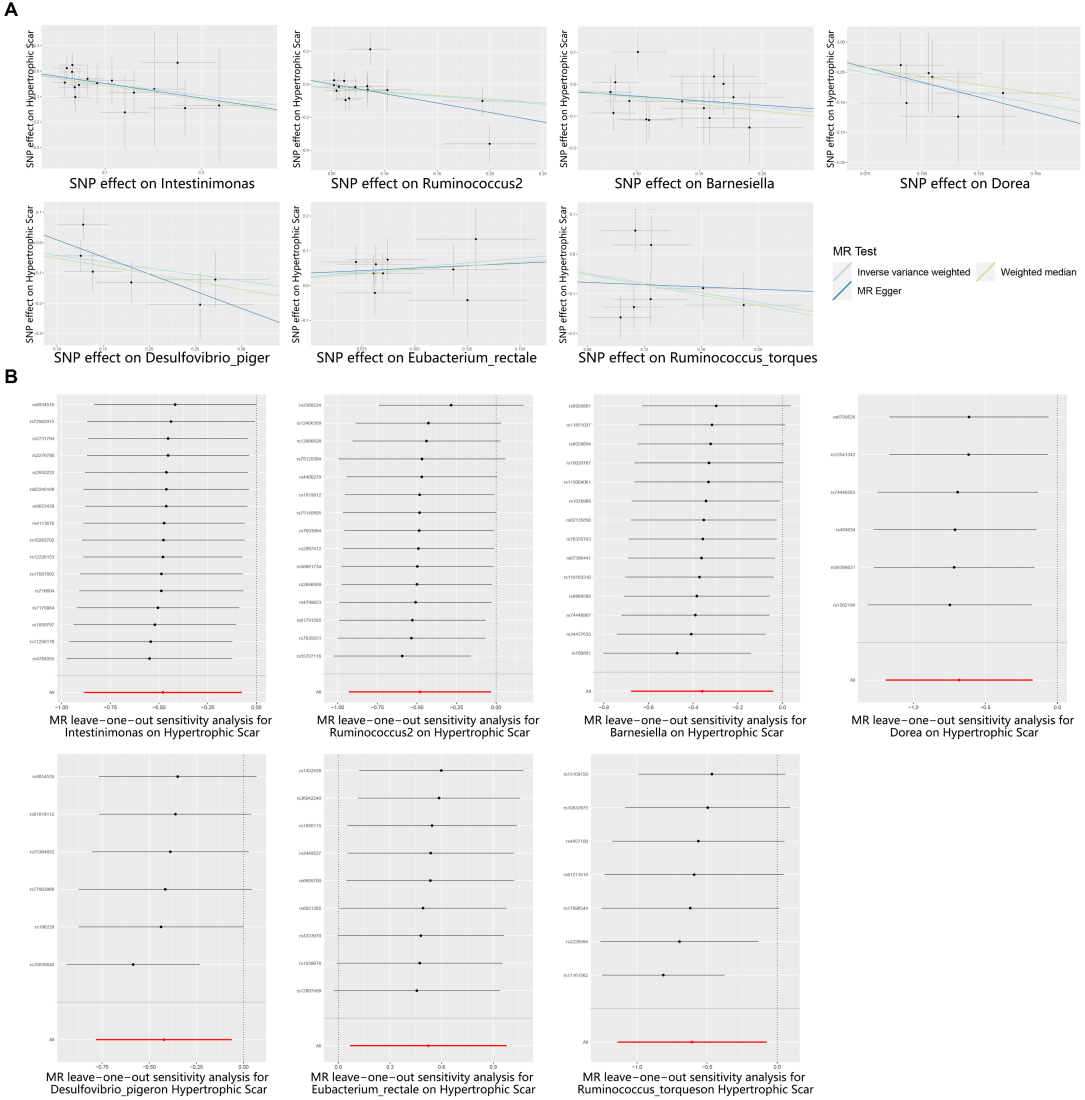
Scatter and Leave-one-out plots for the casual association between gut microbiota and hypertrophic scarring. **(A)** Scatter plots for the causal association between gut microbiota and hypertrophic scarring, and the slope of each line corresponds to the estimated MR effect for each method. **(B)** Leave-one-out plots for the causal association between gut microbiota and hypertrophic scarring. Each black point represents the application of the IVW MR algorithm to estimate the causal effect of gut microbiota on hypertrophic scarring, and the red point represents the IVW estimate using all SNPs.

According to the results provided in Figure 1B (IVW: *p* < 0.05), 11 gut microbiota were identified as being associated with hypertrophic scarring. Following a rigorous screening process, exclusions were made for the unknown genus and gut microbiota that did not meet the specified criteria of instrumental variable selection, as detailed in Supplementary Table S4. As a result, we pinpointed five genera and two species demonstrating associations with hypertrophic scar development (Figure 2). Regarding the genus level, the IVW analysis disclosed that *Intestinimonas* (odds ratio (OR) = 0.62, 95% confidence interval (CI) = 0.41–0.93, *p* = 0.020), *Ruminococcus2* (OR = 0.62, 95% CI = 0.39–0.97, *p* = 0.036), *Barnesiella* (OR = 0.70, 95% CI = 0.51–0.96, *p* = 0.027), *Dorea* (OR = 0.50, 95% CI = 0.30–0.84, *p* = 0.009), and *Desulfovibrio piger* (OR = 0.66, 95% CI = 0.46–0.94, *p* = 0.021) were inversely associated with hypertrophic scarring. At the species level, a positive correlation was observed between *Eubacterium rectale* (OR = 1.69, 95% CI = 1.07–2.66, *p* = 0.024) and hypertrophic scarring, while *Ruminococcus torques* (OR = 0.55, 95% CI = 0.32–0.93, *p* = 0.025) exhibited a negative correlation with hypertrophic scarring. Similarly, analyses employing MR-Egger regression and weighted median (WM) showed that the slope of each line corresponds to the estimated MR effect for each method, with a negative slope indicating a negative correlation and vice versa. The results of MR-Egger and WM methods consistently demonstrated effects in the same direction as the IVW analysis (Figure 3A), suggesting consistency in the results across different MR methods and increasing the robustness of our conclusions. For a comprehensive overview of the results, please refer to the Supplementary Tables S1–S3. Consequently, these findings collectively affirmed a causal link between specific gut microbiota and the incidence of hypertrophic scar.

### 3.2 Sensitivity analysis

To reinforce the purported causal relationships between gut microbiota and hypertrophic scarring, an array of sensitivity analyses was conducted. Heterogeneity was evaluated through leave-one-out analysis and the Q-test of both MR Egger and IVW methods. Leave-one-out plots are a diagnostic tool to assess the influence of individual data points in statistical analyses, and they provide insights into how the removal of specific observations affects the overall results. Each black point represents the application of the IVW algorithm to estimate the causal effect of gut microbiota on hypertrophic scarring, excluding specific variants from the analysis. The red point represents the IVW estimate using all SNPs. Firstly, the leave-one-out analysis revealed no outliers (Figure 3B), and the heterogeneity test confirmed the absence of significant heterogeneities for both IVW and MR Egger models (*p* > 0.05; Supplementary Table S5). Additionally, the Egger intercept, closely approximating zero with *p* > 0.05, indicated no evidence of directional horizontal pleiotropy effects (Supplementary Table S5). In summary, these sensitivity analyses provided robust confirmation of the reliability of our suggested causal effects in the MR results.

### 3.3 Reverse MR analysis

In reverse causality, hypertrophic scarring is used as exposure to validate gut microbiota outcome. The reverse MR analysis did not unveil any potential causality between hypertrophic scarring and the aforementioned bacterial taxa, as indicated by *p* > 0.05 or SNPs <3 (Supplementary Tables S6–S8). The findings indicate that there is no reverse-direction causal relationship between hypertrophic scarring and gut microbiota.

## 4 Discussion

Hypertrophic scar, as a fibroproliferative condition in the reticular dermis layer, is characterized by persistent inflammation, heightened angiogenesis, and excessive collagen deposition (Berman et al., 2017; Ogawa, 2017). Nutritional deficiencies, systemic diseases like diabetes, and autoimmune disorders involving chronic inflammation not only impede the intricate cascade of healing but also foster an environment conducive to the aberrant proliferation of fibroblasts, thereby contributing to the development of hypertrophic scars (Ogawa, 2022; Faour et al., 2023). In fact, the relationship between gut microbiota and skin-associated disorders has been a central focus of extensive investigation in recent years, encompassing conditions like hidradenitis suppurativa, rosacea, acne vulgaris, and atopic dermatitis (Mahmud et al., 2022). Our study unveiled the broadly applicable microbial signatures associated with hypertrophic scarring through performing a bidirectional two-sample MR analysis. Seven causal associations between gut microbiome and hypertrophic scarring were identified, involving *Intestinimonas*, *Ruminococcus2*, *Barnesiella*, *Dorea*, *Desulfovibrio piger*, *Eubacterium rectale*, and *Ruminococcus torques*.

The human gut microbiota is composed of four main phyla, which are collectively known as *Actinobacteria*, *Bacteroidetes*, *Firmicutes*, and *Proteobacteria* (Arumugam et al., 2011). Genus *Barnesiella* from phyla *Bacteroidetes* and genus *Desulfovibrio piger* from phyla *Proteobacteria* exhibited correlations with various immunoregulatory cells, indicating their potential to create a gut environment less susceptible to inflammation (Loubinoux et al., 2002; Weiss et al., 2014). Moreover, in a previous investigation utilizing 16S rRNA gene sequencing, participants with pathological scars exhibited a relative higher abundance of *Firmicutes* (Li M et al., 2023). Similarly, we observed that specific gut microbiota from phyla *Firmicutes* act as protective factors against hypertrophic scarring, including *Intestinimonas*, *Ruminococcus2*, *Dorea*, and *Ruminococcus torques*. Regarding the modified species, *Ruminococcus torques* was reported to be associated with the alleviation of inflammation through regulating bile acid compositions (Zhang M et al., 2023). At the genus level, both *Intestinimonas* and *Ruminococcus2* are butyric acid-producing bacteria, deemed potential probiotics for alleviating and treating inflammatory diseases due to their capacity to regulate T regulatory cells and inhibit Histone Deacetylase (HDAC) (Gao et al., 2023). Additionally, it has been revealed that *Dorea*, a commensal bacterium, functions as immune sentinels within tissues (Zhang et al., 2023b). Moreover, *Ruminococcus* have also been reported to possess the ability to ferment glucose, xylose, and indigestible dietary fiber (Crost et al., 2013; Ghanbari Maman et al., 2020), obtaining energy and nutrients from otherwise complex and difficult-to-digest substrates. Overall, the deficiency of the aforementioned six gut microbiota may contribute to the occurrence of inflammation, representing a potential factor in the onset of hypertrophic scars. On the other hand, the findings concerning the species *Eubacterium rectale* from phylum *Firmicutes* suggested a potential role as a risk factor for hypertrophic scars. This aligns with a prior study indicating that *Eubacterium rectale* contributes to promoting colitis by activating the transcription factor NF-κΒ (Wang et al., 2021). Nevertheless, definitive evidence is necessary to confirm how *Eubacterium rectale* elevates the risk of hypertrophic scarring, given its role as a butyrate-producing flora that generally provides advantages in specific disorders (Lu et al., 2022). To sum up, our study suggested that the mentioned gut microbiota may play a crucial role in the development of hypertrophic scars by modulating the systemic inflammatory response.

In this study, MR analysis was employed to establish the causal relationship between gut microbiota and the development of hypertrophic scar. This approach helped eliminate the impact of confounding variables and minimized the potential for reverse causation, thereby enhancing the ability to infer the causality. Notably, compared to other research (Li Y et al., 2023; Xia et al., 2023; Yang et al., 2023) (211 gut microbiota from MiBioGen consortium), a larger dataset (418 gut microbiota) was employed here through combing GWAS summary statistics from MiBioGen consortium and Dutch Microbiome Project. As a result, a higher statistical power can be achieved and is better at detecting smaller causal effects, providing a more accurate reflection of the likely range of the true causal effect. Furthermore, we identified seven crucial gut microbiotas, from which extracellular vesicles should need further investigation to be a potential nanobiotechnology therapy for hypertrophic scars and wound healing (Han et al., 2023). However, there are several limitations in this study. One notable aspect is that the GWAS summary statistics predominantly stem from European populations, which may affect the generalizability of the findings to other ethnic groups. Besides, while MR analysis demonstrated statistical causality and supported a link between gut microbiota and hypertrophic scarring, further functional experimental research is necessary to validate these results and clarify plausible genetic pathways.

The potential association between a distinct microbiome environment and a disease impacting a disparate physiological tract may be considered plausible. For instance, oral dysbiosis has been linked to an increased risk of cardiovascular diseases. Bacteria associated with periodontal disease can enter the bloodstream, potentially contributing to inflammation and atherosclerosis in blood vessels (Leishman et al., 2010; Teles and Wang, 2011). Besides, emerging research suggested a connection between the gut microbiome and neurodegenerative conditions like Parkinson’s disease. Changes in the gut microbiome composition might influence the development and progression of neurodegenerative diseases through the gut-brain axis (Zhu et al., 2021; Zhang et al., 2023a). From a similar perspective, this study analyzed the impact of the non-cutaneous microbial environment on hypertrophic scarring. However, the evidence about this microbiome influence is still unclear or inconsistent. On the contrary, the evidence supporting the influence of local microbiota on the skin is continually growing. Changes in the skin microbiome have been implicated in various skin conditions, such as seborrheic dermatitis and acne (Ferček et al., 2021). It has also been found that the dysbiosis of the microbiota occurring in hypertrophic scars is primarily associated with *S. aureus* colonization (Yu et al., 2023). Currently, there is a lack of research on the influence of gut microbiota on skin microbiota and the impact of skin microbiota on hypertrophic scars. Further research is needed to explore these aspects.

## 5 Conclusion

In this study, we conducted a comprehensive assessment of the causal connections between gut microbiota and the development of hypertrophic scar. Through MR analysis, six bacterial taxa were identified as protective factors, while one was identified as a risk factor for hypertrophic scar. In particular, the reverse MR study failed to demonstrate a reverse causal relationship between gut microbiota and hypertrophic scarring. Our study contributed additional supportive evidence and valuable insights into the causal relationship between gut microbiota and the development of hypertrophic scar, providing avenues for mechanistic exploration and the identification of potential therapeutic targets.

## Data availability statement

The original contributions presented in the study are included in the article/Supplementary material, further inquiries can be directed to the corresponding authors.

## Author contributions

KX: Conceptualization, Methodology, Software, Validation, Writing – original draft, Writing – review & editing. GZ: Data curation, Formal analysis, Investigation, Resources, Writing – original draft. ZihL: Data curation, Formal analysis, Investigation, Resources, Writing – original draft. XZe: Data curation, Formal analysis, Investigation, Resources, Writing – review & editing. ZiL: Visualization, Writing – review & editing. FW: Visualization, Writing – review & editing. XZh: Visualization, Writing – review & editing. CL: Supervision, Validation, Visualization, Writing – review & editing. CM: Supervision, Validation, Visualization, Writing – review & editing.

## Footnotes

^1^http://zzz.bwh.harvard.edu/plink/

^2^https://github.com/WMU-kk/MR-GM-HS
